# The Effect of Oleanolic Acid and Its Four New Semisynthetic Derivatives on Human MeWo and A375 Melanoma Cell Lines

**DOI:** 10.3390/ph16050746

**Published:** 2023-05-14

**Authors:** Barbara Bednarczyk-Cwynar, Anna Leśków, Izabela Szczuka, Lucjusz Zaprutko, Dorota Diakowska

**Affiliations:** 1 Department of Organic Chemistry, Poznan University of Medical Science, Grunwaldzka 6, 60-780 Poznan, Poland; bcwynar@ump.edu.pl (B.B.-C.); zaprutko@ump.edu.pl (L.Z.); 2 Department of Basic Sciences, Wroclaw Medical University, Chalubinskiego 3, 50-368 Wroclaw, Poland; 3 Department of Biochemistry and Immunochemistry, Wroclaw Medical University, Chalubinskiego 10, 50-368 Wroclaw, Poland; izabela.szczuka@umw.edu.pl

**Keywords:** triterpenes, oleanolic acid derivatives, human melanoma, cytotoxicity

## Abstract

This study aimed to synthesize four new semisynthetic derivatives of natural oleanolic acid (OA) and, based on an analysis of their cytotoxic and anti-proliferative effects against human MeWo and A375 melanoma cell lines, select those with anti-cancer potential. We also screened the treatment time with the concentration of all four derivatives. We synthesized oxime 2 and performed its acylation with carboxylic acids into new derivatives **3a, 3b, 3c** and **3d** according to the methods previously described. Colorimetric MTT and SRB assays were used to measure the anti-proliferative and cytotoxic activity of OA and its derivatives **3a**, **3b**, **3c** and **3d** against melanoma cells. Selected concentrations of OA, the derivatives, and different time periods of incubation were used in the study. The data were analyzed statistically. The present results revealed the possible anti-proliferative and cytotoxic potential of two selected OA derivatives **3a** and **3b**, on A375 and MeWo melanoma cells, especially at concentrations of 50 μM and 100 μM at 48 h of incubation (*p* < 0.05). Further studies will be necessary to analyze the proapoptotic and anti-cancer activities of **3a** and **3b** against skin and other cancer cells. The bromoacetoxyimine derivative (**3b**) of OA morpholide turned out to be the most effective against the tested cancer cells.

## 1. Introduction

Human skin is the organ most exposed to environmentally damaging and mutagenic factors, which may result in cancer formation. The most common skin cancers are basal cell carcinoma and squamous cell carcinoma [1,2,3], although malignant melanoma is considered the most deadly, being responsible for 90% of deaths associated with cutaneous tumors [4]. Melanoma is caused by the abnormal proliferation of melanocytes [5]. These pigment-producing cells are present in the basal layer of the epidermis. Melanocytes are derived from a neural crest origin and produce many signaling molecules and other bioactive factors that promote migration and metastasis after malignant transformation [5,6]. Most types of malignant melanoma are believed to be caused by sun (UV) exposure without protection, although gender (men), age (above 65 years old), family history, genetic predispositions, indoor tanning, moles or nevi, and obesity may contribute to this disease [4,5,6]. In recent years, there has been an increase in malignant melanoma in white men over 60 years of age in Sweden and northern Europe [4,6,7]. An increase in melanoma incidence was also observed in all age groups in other parts of Europe [4,7], and this situation has not stabilized [6,7]. Primary melanoma usually appears in a cutaneous location, although this tumor metastasizes quickly [8,9]. Malignant melanoma can have several systemic consequences. After it spreads to other areas of the body, such as the lungs, liver, brain, and bones, metastatic melanoma causes damage to these organs and affects their normal functioning. The consequences of metastatic melanoma depend on the location and extent of the metastases. For example, if melanoma spreads to the liver, it can cause abdominal pain, nausea, and jaundice [10]. In addition to the physical consequences, metastatic melanoma can also have significant psychological effects on patients and their families, including anxiety, depression, and decreased quality of life [11]. The rapid spread of melanoma to different organs can make effective treatment challenging or even impossible.

The treatment of melanoma is difficult and depends on the stage of cancer progression and subtype. Operative treatment (tumor resection), radiotherapy, chemotherapy, adjuvant and neoadjuvant therapy or systemic therapy for metastatic disease represent the current standard approaches for managing this highly aggressive disease [12]. The systemic treatment of melanoma may be hindered by the development of multidrug resistance [5,13]. Patient survival rates are significantly impacted by factors such as the rate of metastasis, as well as the number and location of metastases [3,14,15]. Thus, there is a need for strengthening the effect of anti-cancer drugs by developing compounds that support chemotherapy [16,17] and slow the process of metastasis.

Currently, for the advancement of treatment options, natural-derived compounds with anti-proliferative and anti-metastatic properties are being investigated for chemoprevention and a reduction in side effects in the treatment of melanoma. Among them, triterpenoid compounds are an important class of phytochemicals with pro-apoptotic, anti-proliferative and tumor anti-invasive potential [16,17,18]. Many studies have indicated that OA, belonging to the pentacyclic triterpenes, and its derivatives are molecules that possess numerous pharmacological activities against malignant tumor development [16,17,19]. OA exerts anti-cancer effects in thyroid, rectal, colon, liver, gastric, lung, and breast malignancy through influencing several molecular mechanisms including: inhibition of signaling pathways that stimulate cancer cells development, survival, differentiation, and proliferation, as well as oncogenic growth factors and cell cycle proteins expression; the induction of pro-apoptotic pathways; stimulation of the reactive oxygen species in cancer cells with the autophagy process facilitation [16,17,18,19,20]; and the production of antioxidant enzymes [21]. Experimental studies indicated that OA acts as a pro-apoptotic compound on A375 melanoma cell lines [22,23], showing anti-proliferative activity against human melanoma WM-266-4 cell lines [24], SK-MEL-2 melanoma cells [18], and A375 and B16 4A5 melanoma cell lines [22]. The OA derivatives exert autophagic potential and decreased breast cancer cells migration [25], inducing apoptosis in hepatoma cells [26] and melanoma cells [27]. There are many papers that describe the relationship between the structure of triterpenes and their biological activity. Moreover, the hydroxyimin group in position 3 of triterpenes, especially of OA, is responsible for an increase in anti-cancer activity [28]. In addition, the transformation of the C-28 carboxyl group into an amide has a more positive effect on the pharmaceutical properties of the modified molecule than its modification into the ester group [29]. Among the amides, the morpholides and the imidazolides seem to be the most effective anticancer agents [30]. Therefore, we synthesized four new semisynthetic OA derivatives with these moieties.

The aim of our study was to synthesize four new OA derivatives and define their anti-cancer potential, especially their cytotoxicity and anti-proliferative effect on human MeWo and A375 melanoma cell lines. The additional objective of our study was to select the most promising compounds and determine their most effective concentrations.

## 2. Results

### 2.1. Synthesis of Cytotoxic Agents

To explore the effects of substituents within the acyloxyimino group on cytotoxic activity, the C-3 hydroxyl group was converted into oxo, and then into hydroxyimino and acyloxyimino groups. As previously shown, the transformation of the C-17 carboxyl group into morpholide moiety leads to an improvement of cytotoxic activity of OA derivatives, which is why the second transformation of the OA structure was the introduction of a morpholide ring instead of hydroxyl within the -COOH function [30].

**Figure 1:**
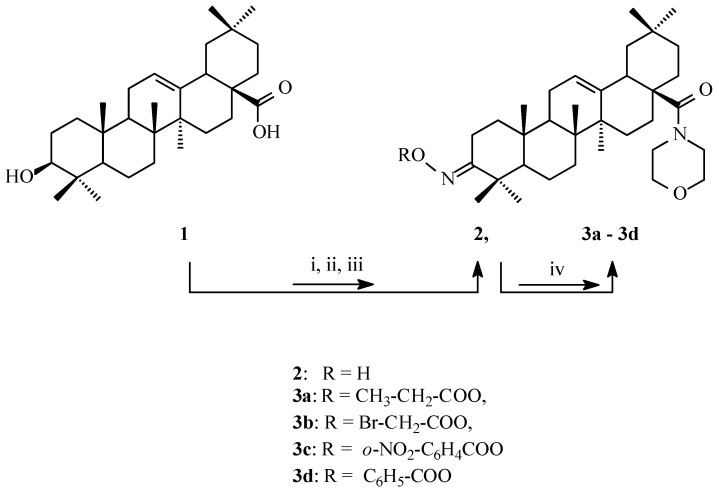
The synthesis procedure of anticancer agents. OA (1), oxime (2), acylated oximes of OA morpholide (**3a**–**3d**). i: Jones reagent, acetone, rt.; ii: (COCl_2_), rt.; morpholine, benzene, rt.; iii: NH_2_OH x HCl, CH_3_COONa, ethanol, reflux, iv: dioxane, DCC, (Ar)RCOOH, rt.

Figure 1 shows that OA (1) was the starting material for performing our syntheses [31]. The obtained oxime (2) was acylated by using a procedure known from the literature data [31]. As a result, four acylated oximes of OA morpholide (**3a**–**3d**) were obtained, with the carboxylic group at the C-17 position transformed into an amide system. The structures of the obtained acylated oximes were confirmed with the spectral data (^1^H NMR, ^13^C NMR, DEPT).

### 2.2. Effects of OA and Its Derivatives on the Viability of Human MeWo Cells—MTT and SRB Assays

**Figure 2:**
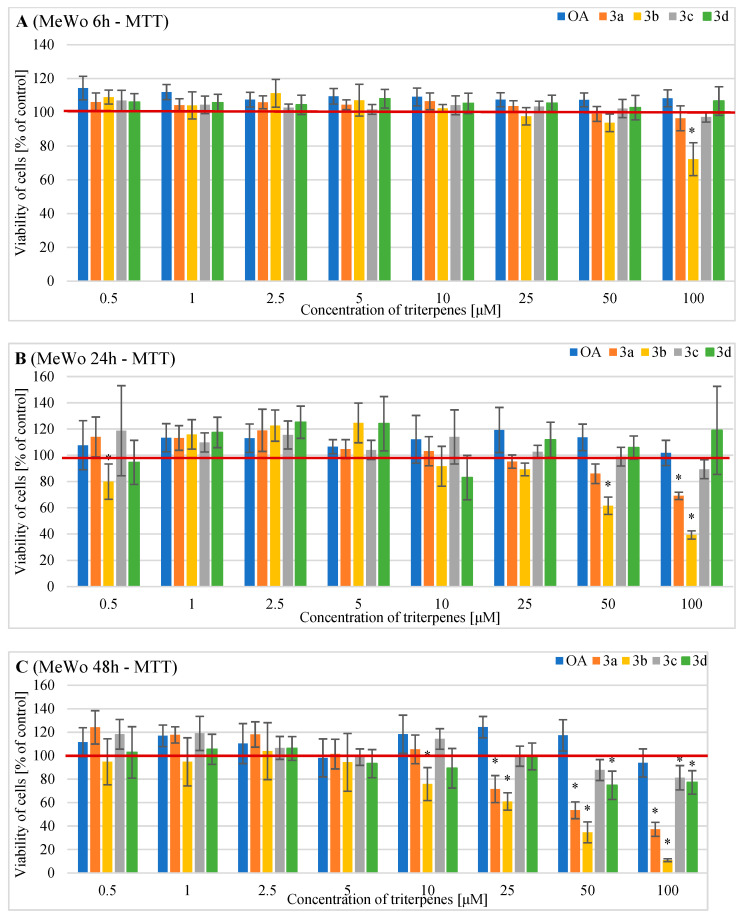
The results of the MTT assay (the metabolic activity test) on the viability of MeWo cells incubated with triterpenes OA, **3a**, **3b**, **3c,** or **3d** for (**A**) 6 h, (**B**) 24 h or (**C**) 48 h. Descriptive data were presented as mean ± SD. Statistically significant differences versus control cells were marked with an asterisk (* *p* ≤ 0.05). The horizontal bold line represents the viability of control (untreated) cells.

The effects of selected OA concentrations and its derivatives on the viability of the human MeWo melanoma cells were evaluated following a treatment period of 6 h, 24 h and 48 h using an MTT assay (Figure 2).

OA and its derivatives **3a, 3c,** and **3d** at each concentration did not exert any cytotoxic activity after 6 h of incubation. Derivative **3b** at 100 µM reduced MeWo cell viability to 72.2% (Figure 2A).

After 24 h, derivative **3a** at the highest tested concentration of 100 µM significantly decreased cell viability to 69.1%, while derivative **3b** significantly decreased cell viability to 61.5% and 39.2% at 50 and 100 µM, respectively. We did not observe significant cytotoxic effects for the remaining tested derivatives after 24 h of incubation (Figure 2B).

After 48 h of treatment with triterpenes, MeWo melanoma cell viability was significantly reduced at the following compound concentrations: 71.6% for **3a** at 25 µM, 53.5% for **3a** at 50 µM and 37.2% for **3a** at 100 µM; 81.2% for **3c** at 100 µM; 75.8% for **3b** at 10 µM, 61% for **3b** at 25 µM, 34.7% for **3b** at 50 µM and 10.9% for **3b** at 100 µM; 74.8% for **3d** at 50 µM and 77.2% for **3d** at 100 µM (Figure 2C). There was no statistically significant reduction in cell survival after OA treatment.

**Figure 3:**
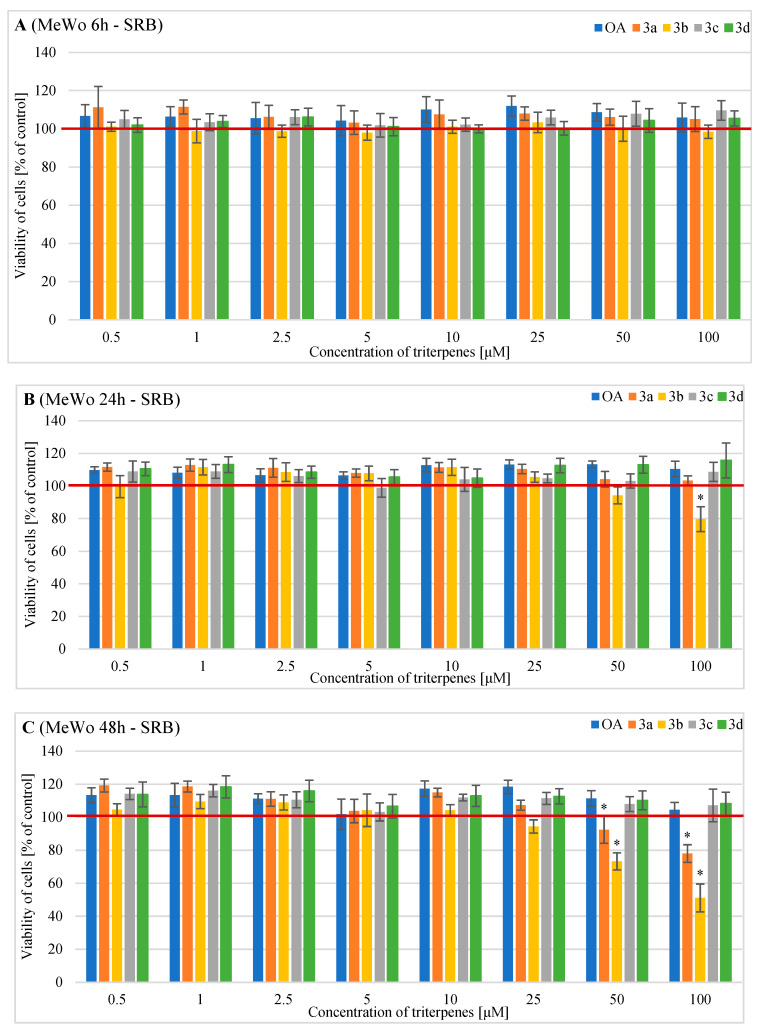
The results of the SRB assay (the cell number test) on the viability of MeWo cells incubated with triterpenes OA, **3a**, **3b**, **3c,** or **3d** for (**A**) 6 h, (**B**) 24 h or (**C**) 48 h. Descriptive data were presented as mean ± SD. Statistically significant differences versus control cells were marked with an asterisk (* *p* ≤ 0.05). The horizontal bold line represents the viability of control (untreated) cells.

In the SRB test (Figure 3), no statistically significant reduction in human MeWo cell viability was demonstrated after 6 h of treatment with different concentrations of OA and its derivatives (Figure 3A).

After 24 h incubation, only derivative **3b** at 100 µM significantly reduced cell viability to 79.6% (Figure 3B).

After 48 h of treatment, derivative **3a** showed a significant cytotoxic effect on MeWo cells at 50 µM (92.4% viable cells) and 100 µM (78% viable cells) (Figure 3C). Derivative **3b** at 50 µM significantly decreased MeWo cell viability to 73.2%, and at a concentration of 100 µM, it significantly reduced viability to 51.1% (Figure 3C).

### 2.3. Effects of OA and Its Derivatives on Viability of Human A375 Cells—MTT and SRB Assays

**Figure 4:**
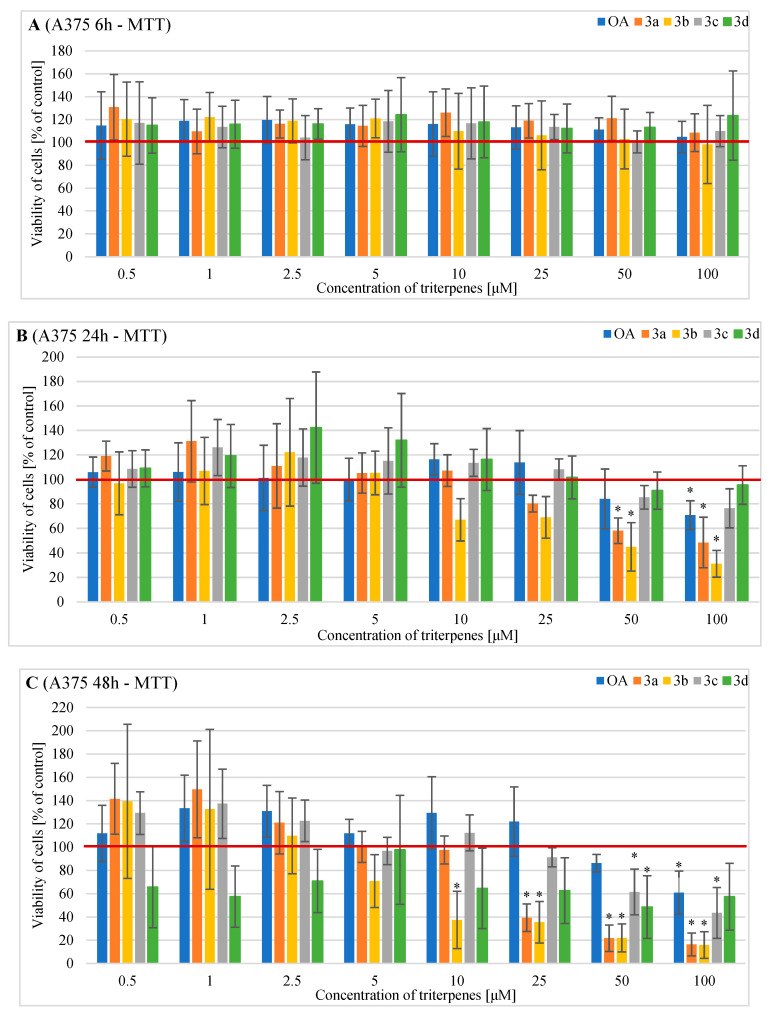
The results of the MTT assay (the metabolic activity test) on the viability of A375 cells incubated with triterpenes OA, **3a**, **3b**, **3c,** or **3d** for (**A**) 6 h, (**B**) 24 h or (**C**) 48 h. Descriptive data were presented as mean ± SD. Statistically significant differences versus control cells were marked with an asterisk (* *p* ≤ 0.05). The horizontal bold line represents the viability of control (untreated) cells.

**Figure 5:**
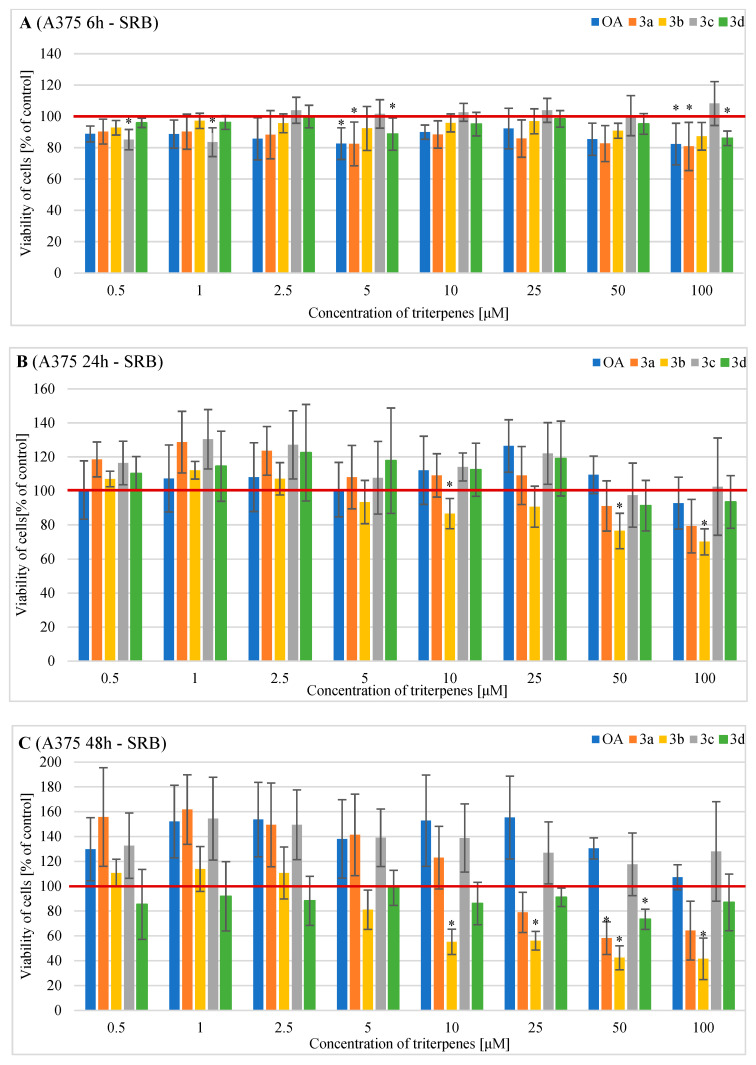
The results of the SRB assay (the cell number test) on the viability of A375 cells incubated with triterpenes OA, **3a**, **3b**, **3c**, or **3d** for (**A**) 6 h, (**B**) 24 h or (**C**) 48 h. Descriptive data were presented as mean ± SD. Statistically significant differences versus control cells were marked with an asterisk (* *p* < 0.05). The horizontal bold line represents the viability of control (untreated) cells.

The effects of OA and its derivatives on the viability of the human A375 melanoma cells were evaluated following a treatment period of 6 h, 24 h and 48 h using MTT and SRB assays (Figure 4 and Figure 5).

No statistically significant reduction in human A375 cell viability was demonstrated after 6 h treatment with different concentrations of triterpenes (Figure 4A).

After 24 h treatment, cell viability was significantly reduced by OA at 100 µM to 70.8%, derivative **3a** at 25 µM decreased cell viability to 58.1%, and **3a** at 100 µM reduced cell viability to 48.5% (Figure 4B). Derivative **3b** significantly decreased human A375 cell viability, as follows: 45% at 50 µM and 31.2% at 100 µM (Figure 4B).

After 48 h of treatment, derivative **3a** at 25 µM showed a significant cytotoxic effect on A375 melanoma cells (39.4% viable cells), as well as at 50 µM (21.7% viable cells) and at 100 µM (16.4% viable cells) (Figure 4C). Additionally, derivative **3b** significantly decreased human A375 cell viability, as follows: at 10 µM to 37.5%, at 25 µM to 35.5%, at 50 µM to 22% and at 100 µM to 16% (Figure 4C). OA alone and its derivatives **3c** and **3d** reduced A375 cell viability to a lesser extent. OA at 100 µM significantly reduced A375 cell viability to 61%, derivative **3c** at 50 and 100 µM significantly decreased cell viability to 61.5% and 43.5%, respectively, and derivative **3d** at 50 µM significantly reduced cell viability to 48.6% (Figure 4C).

The results observed in the SRB were varied. After 6 h of treatment, derivative **3a** significantly reduced the viability of A375 melanoma cells under the following conditions: at 5 µM to 82.5% and at 100 µM to 80.9%. Derivative **3c** at 0.5 and 1 µM decreased cell viability to 85.2% and 83.5%, respectively. No cytotoxic effect was observed at higher concentrations of **3c** (Figure 5A). Derivative **3d** at 5 and 100 µM µM decreased cell viability to 88.7% and 86.0%, respectively. A similar effect was observed for OA, which at concentrations of 5 and 100 µM decreased cell viability to 82.6% and 82.4%, respectively (Figure 5A).

After 24 h of treatment, only derivative **3b** significantly reduced the viability of A375 melanoma cells, as follows: at 10 µM to 86.6%, at 50 µM to 76.5%,and at 100 µM to 70.1% (Figure 5B).

After 48 h of treatment, derivative **3b** significantly decreased A375 cell viability at the following concentrations: at 10 µM to 55.2%, at 25 µM to 56.1%, at 50 µM to 42.5%, and at 100 µM to 41.6% (Figure 5C). Derivatives **3a** and **3d** at a concentration of 50 µM decreased cell viability to 58.2% and 73.4%, respectively (Figure 5C). OA and its derivative **3c** did not exert any cytotoxic effect after 48 h of incubation (Figure 5C).

## 3. Discussion

Chemotherapy, targeted therapy and immune checkpoint inhibitor therapy are more effective after the surgical excision of the primary tumor at any stage of cutaneous melanoma [5,12]. Additionally, synthetic drugs usually act equally on normal and tumor cells, which may cause more harm than selective drugs [16,17]. The combination of therapeutic efficiency with the lack of toxic activity makes biologically active compounds, such as triterpenes, an attractive alternative to conventional treatment [16,17]. One of the best-known representatives of pentacyclic triterpenes is OA, which exhibits anti-cancer effects on human carcinoma cell lines. OA participates in blocking the migration and invasion of thyroid cancer cells and hepatocellular carcinoma cells [32,33]. Moreover, OA inhibits rectal cells proliferation [34] and induces autophagy and apoptosis in colon cancer cells and hepatoma cells [35,36]. However, studies have confirmed that OA is characterized by poor solubility in water, which limits its use in pharmacotherapy [25,37]; however, it can be successfully modified to improve its broad activity and bioavailability. Several OA derivatives, acylated in various manners in the 3-hydroxyimino group, have been described in numerous research articles. They all showed higher anti-cancer activity than the starting OA. Here, we prepared four new OA derivatives, which exhibited better anti-proliferative and cytotoxic effects on human melanoma cell lines; furthermore, possibly, due to the presence of a morpholine moiety at the C-28 position, they have more advantageous characteristics than other similar derivatives. Here, as a result of our research, the effect of the acyl group’s structure at the C-3 position of the 3-hydroxyimino OA derivatives on the anti-proliferative activity of the obtained new compounds was determined.

We tested the OA and its derivatives to determine its cytotoxic effects against human MeWo and A375 melanoma cell lines with an SRB assay, and the anti-proliferative properties of substances were measured with an MTT assay. The cells were incubated with triterpenes for 6 h, 24 h and 48 h.

The results of the MTT assay showed that the **3b** derivative exerts a significant dose- and time- dependent anti-proliferative effect in both melanoma cell lines. Analyzing the six-hour incubation time, in the case of MeWo cells, only the **3b** derivative at a concentration of 100 µM showed a significant anti-proliferative effect on the tested cells. Due to the longer incubation time, the **3b** derivative already significantly inhibited MeWo cell proliferation at a minimum concentration of 10 µM. The SRB assay used at the same incubation times and with the same concentrations of the **3b** substance showed a slightly weaker cytotoxic effect on MeWo cells. In this case, a statistically significant reduction in cell viability was demonstrated for a concentration of 50 µM after 48 h incubation.

The second compound demonstrating a significant anti-proliferative effect on MeWo cells was the **3a** derivative. The MTT assay showed its statistically significant inhibition of cell viability after 48 h of incubation and at a minimum concentration of 25 µM. In the case of the SRB assay, the cytotoxicity of the **3a** derivative was also demonstrated after 48 h of incubation, but at a minimum concentration of 50 µM. Derivatives **3c** and **3d** exerted cytotoxic activity after 48 h of incubation and at concentrations of 50 µM and 100 µM only in the MTT assay. We demonstrated that OA did not induce any changes in the MeWo cell viability regardless of the tested concentrations.

Ours is the first study to evaluate the cytotoxicity of OA derivatives on MeWo cell lines; therefore, our obtained data cannot be compared with other studies. Welch et al. [30] reported that the MeWo melanoma cell line is characterized by a moderate metastatic potential compared with other melanoma cell lines, for example A375. It has been shown that cells of the MeWo line have exhibited prediction to extrapulmonary metastases.

OA and its derivatives were also tested on the A375 melanoma cell line. Again, the most promising results in the MTT assay were obtained for the **3a** and **3b** derivatives. The application of the 50 µM sample concentration with 24 h incubation, and the cell treatment of a minimum of 10 µM for **3b** or 25 µM for **3a** within 48 h, induced significant decreases in cell viability. Cytotoxic effects also occurred for the **3b** derivative at a minimum of 10 µM and 24 h or 48 h of incubation in the SRB assay, whereas derivative **3a** demonstrated a cytotoxic effect after 6 h of incubation at a concentration of 100 µM or at 50 µM and 48 h of incubation in the SRB assay. Derivatives **3c** and **3d** showed anti-proliferative activity at a concentration of 50 µM and 48 h of incubation in the MTT assay. Only the **3d** derivative significantly decreased cell viability at 100 µM and 6 h of incubation or at 50 µM and 48 h of incubation. Our findings confirmed that 3-hydroxyimine derivatives of OA morpholides show anti-cancer potential higher than the starting and comparative unmodified OA. Moreover, the acylation of the hydroxyimine group may have a beneficial cytotoxic effect in the used cell lines. Comparing the effectiveness of new compounds with various types of acyl substituents, we concluded that the highest efficiency was shown by products with an aliphatic substituent, especially with an electron-withdrawing substituent.

In the case of the A375 cell line, OA exerts a cytotoxic effect at a concentration of 100 µM and 24 h or 48 h of incubation (MTT assay). However, the SRB assay did not show a significant inhibition of cell viability after the application of each OA concentration. The results obtained by Mioc et al. [23] confirmed that OA at 50 µM and a treatment period of 24 h significantly decreased A375 melanoma cell viability to 74.8%. They also tested benzotriazole ester of OA, which showed a better dose-dependent reduction in cell viability against A375 cells and no cytotoxic effect against healthy human keratinocytes. Additionally, Oprean et al. [22] demonstrated that OA exerts a significant cytotoxic effect on A375 melanoma cells at 50 µM and 48 h of incubation (78% cell viability), although this effect was determined to be low to moderate compared with the cytotoxic activity of ursolic acid (UA). Research conducted on the SK-MEL-2 melanoma cell line demonstrated that UA exerted a significant dose-dependent anti-proliferative effect in vitro compared with OA. Further testing the two compounds as a mixture is needed to reveal the possible synergic or additive effects on blood vessels and tumor cells [18]. Isakovic-Vidovic et al. [24] reported the promising inhibitory effects of OA mixtures with betulinic acid and UA on WM-266-4 metastatic melanoma cells proliferation activity.

The present results reveal the possible anti-proliferative and cytotoxic potential of two selected OA derivatives, namely **3a** and **3b**, on A375 and MeWo melanoma cells. Further studies will be necessary to analyze the pro-apoptotic and anti-cancer activities of the **3a** and **3b** derivatives against kin and other cancer cells. The bromoacetoxyimine derivative of OA morpholide was the most effective against the tested cancer cells.

In summary, we synthesized four new OA derivatives and determined that two of them, **3a** and **3b**, showed significant anti-cancer activity against the MeWo and A375 melanoma cell lines. This indicates that alkyl derivatives are preferable to aryl ones in the present experiment. In addition, we determined that the most effective concentrations for **3a** are 50 and 100 µM at 48 h for both tested cell lines, while for derivative **3b** it is 100 µM with 24 h of incubation and 50 µM, and 100 µM with 48 h of incubation for both tested cell lines.

In the future, we are planning to study appropriately selected compounds on other melanoma cells and normal cells, including fibroblasts and keratinocytes, because melanoma mainly proliferates in the skin.

## 4. Materials and Methods

### 4.1. General Synthesis Procedure for Derivatives ***3a***, ***3b***, ***3c*** and ***3d***

- General information

All commercially available solvents and reagents used in our experiments were graded “pure for analysis” (Chempur or Sigma-Aldrich). The solvents were dried according to the usual procedures. Products were purified by column chromatography using 70–230 mesh silica gel (Merck). TLC analysis was performed with the application of benzene and ethyl acetate in given volume ratios. Melting points were measured with a Büchi apparatus in an open capillary and are uncorrected. Elemental analyses (C, H, N) were performed with a Perkin-Elmer 2400 CHN analyzer. **^1^**H and **^13^**C NMR spectra were recorded on a Varian Gemini 300 VT spectrometer for frequencies of 300 MHz and 75 MHz, respectively. TMS (δ = 0 ppm) was used as an internal standard. The multiplicity are reported as follows: s = singlet, d = doublet, t = triplet, qu = quartet, m = multiplet, dd = doublet doublets, dt = doublet triplets, br/s = broad singlet. ESI-MS spectra were recorded on a QTOF (5600+, AB Sciex) spectrometer.

- Synthesis of oximes 2

Synthesis of oxime 2 was performed according to methods known from literature data [38]. Physical and spectral data were in agreement with those from literature data [38].

- Acylation of oxime 2 with carboxylic acids

Acylation of oxime 2 with carboxylic acids was performed according to methods known from literature data [38] starting from 1.0 mmol of each oxime.

3-Propionoxyimino-olean-12-en-28-oic acid morpholide (comp. **3a**): C**_37_**H**_58_**N**_2_**O**_4_**. Mol. mass: 594.88. Yield: 535 mg (89.9%). M.p.: 105–110 °C (precipt. with H**_2_**O from EtOH sol., white powder). Rf: 0.74 (2:1), 0.56 (4:1), 0.24 (9:1). **^1^**H NMR (δ, ppm): 5.27 (**^1^**H, t, J = 3.7 Hz, C12-H); 3.71–3.60 (8H, m, -COMorph); 3.08 (1H, d, J = 11.0 Hz, C18-Hβ); 2.47 (2H, qu, J = 7.5 Hz, CH**_3_**-CH**_2_**-COON=C<); 1.16, 1.14, 1.12, 1.03, 1.02, 0.93, 0.77 (7 × 3H, 7 × s, 7 × CH**_3_**); 1.21 (3H, t, J = 7.5 Hz, CH**_3_**-CH**_2_**-COON=C<). **^13^**C NMR (δ, ppm): 176.1 (Cq, -COMorph); 174.8 (Cq, C-3); 172.9 (Cq, CH**_3_**-CH**_2_**-COON=C<); 144.8 (Cq, C-13); 121.3 (CH, C-12); 66.9 × 2, 46.0 and 41.9 (4 × CH**_2_**, -COMorph); 46.3 (Cq, C-17); 27.2 (CH**_2_**, CH**_3_**-CH**_2_**-COON=C<); 9.0 (CH**_3_**, CH**_3_**-CH**_2_**-COON=C<). DEPT: 8 × CH**_3_**, 15 × CH**_2_**, 4 × CH, the total number of C atoms: 37.

3-Bromoacetoxyimino-olean12-en-28-oic acid morpholide (comp. **3b**): C**_36_**H**_55_**BrN**_2_**O**_4_**. Mol. mass: 658.33. Yield: 548 mg (83.3%). M.p.: 123–130 °C (precipt. with H**_2_**O from EtOH sol., white powder). Rf: 0.74 (2:1), 0.54 (4:1), 0.22 (9:1). **^1^**H NMR (δ, ppm): 5.28 (1H, d, J = 3.7 Hz, C12-H); 4,00 (2H, s, Br-CH**_2_**-COON=C<); 3.71–3.61 (8H, m, -COMorph); 3.08 (1H, d, J = 11.0 Hz, C18-Hβ); 1.14, 1.13, 1.04, 1.02, 0.93, 0.90 (7 × 3H, 7 × s, 7 × CH**_3_**). **^13^**C NMR (δ, ppm): 176.3 (Cq, -COMorph); 175.2 (Cq, C-3); 175.2 (Cq, Br-CH**_2_**-COON=C<); 144.7 (Cq, C-13); 121.5 (CH, C-12); 66.9 × 2, 46.0 and 41.9 (4 × CH**_2_**, -COMorph); 46.3 (Cq, C-17); 25.8 (Cl-CH**_2_**-COON=C<). DEPT: 7 × CH**_3_**, 15 × CH**_2_**, 4 × CH, total number of C atoms: 36.

3-(2**′**-Nitro)benzoxyimino-olean-12-en-28-oic acid morpholide (comp. **3c**): C**_41_**H**_57_**N**_3_**O**_6_**. Mol. mass: 687.92. Yield: 614 mg (89.3%). M.p.: 109–115 °C (precipt. with H**_2_**O from EtOH sol., yellowish powder). Rf: 0.72 (2:1), 0.58 (4:1), 0.24 (9:1). **^1^**H NMR (δ, ppm): 8.02 (1H, d, J = 7.7 Hz) and 7.44–7.72 (3 × 1H, m, 2**′**-NO**_2_**-Ar-COON=C<); 5.27 (1H, t, J = 3.7 Hz, C12-H); 3.71–3.61 (8H, m, -COMorph); 3.08 (1H, d, J = 12.0 Hz, C18-Hβ); 1.17, 1.13, 1.10, 1.08, 0.93, 0.90, 0.77 (7 × 3H, 7 × s, 7 × CH**_3_**). **^13^**C NMR (δ, ppm): 176.4 (Cq, -COMorph); 175.2 (CH, C-3); 162.3 (Cq, 2**′**-NO**_2_**-Ar-COON=C<); 147.5 (Cq), 133.3 (CH), 131.3 (CH), 129.8 (CH), 123.7 (Cq) and 124.4 (CH, 3**′**-NO**_2_**-Ar-COON=C<); 144.7 (Cq, C-13); 121.3 (CH, C-12); 66.9 × 2, 46.3 and 41.9 (4 × CH**_2_**, -COMorph); 46.3 (Cq, C-17). DEPT: 8 × CH**_3_**, 14 × CH**_2_**, 8 × CH, total number of C atoms: 38.

3-Benzoxyiminoolean-12-en-28-oic acid morpholide (comp. **3d**): C**_41_**H**_58_**N**_2_**O**_4_**. Mol. mass: 642.92. Yield: 609 mg (94.7%). M.p.: 108–112 °C (precipt. with H**_2_**O from EtOH sol., white powder). Rf: 0.76 (2:1), 0.59 (4:1), 0.47 (9:1). **^1^**H NMR (δ, ppm): 8.05 (2H, dd, J = 7.3 and 1.2 Hz) and 7.60 (1H, tt, J = 7.4 and 1.2 Hz) and 7.47 (2H, tt, J = 7.7 and 1.3 Hz, Ar-COON=C<); 5.28 (1H, t, J = 3.7 Hz, C12-H); 3.71–3.64 (8H, m, -COMorph); 3.09 (1H, d, J = 11.0 Hz, C18-Hβ); 1.15, 1.14, 1.13, 1.06, 0.93, 0.90, 0.79 (7 x 3H, 7 x s, 7 x CH**_3_**). **^13^**C NMR (δ, ppm): 176.2 and 176.1 (2 × Cq, -COMorph and C-3); 164.5 (Cq, Ar-COON=C<); 144.8 (Cq, C-13); 133.1, 129.5 × 2, 128.2 × 2 (5 × CH) and 126.7 (Cq, Ar-COON=C<); 121.2 (CH, C-12); 66.9 × 2, 46.0 and 41.9 (4 × CH**_2_**, -COMorph); 46.3 (Cq, C-17). DEPT: 7 × CH**_3_**, 14 × CH**_2_**, 9 × CH, total number of C atoms: 38.

### 4.2. Cell Culture

Two certified human malignant melanoma cell lines: A375 (ATCC**^®^** CRL-1619™) and MeWo (ATCC**^®^** HTB-65™) were obtained from the American Type Culture Collection (ATCC; Manassas, VA, USA) and were cultured in a complete growth medium that contained Dulbecco’s Modified Eagle Medium without phenol red (DMEM; Gibco, Thermo Fisher Scientific, Waltham, MA, USA) supplemented with 10% heat-inactivated fetal bovine serum (FBS; Gibco, Thermo Fisher Scientific, Waltham, MA, USA) with addition of stabilized 1% antibiotic antimycotic solution containing 25 µg/mL of amphotericin B, 10,000 units/mL of penicillin, and 10 mg/mL of streptomycin (Sigma-Aldrich, St. Luis, MO, USA). Medium was renewed every 3 days. Cells were cultured in a CELCULTURE**^®^** CCL-170B-8 CO**_2_** incubator (Esco, Singapore) at 37 °C in 95% humidified air with 5% CO**_2_**. Cells were harvested from cell culture T-75 flasks (Eppendorf AG, Hamburg, Germany) with TrypLE™ Express (Gibco, Thermo Fisher Scientific, Waltham, MA, USA), stained with 0.4% trypan blue solution and counted with Countess™ Automated Cell Counter (Invitrogen, Thermo Fisher Scientific, Waltham, MA, USA). For the experiments, the cells were grown on 96-well tissue-culture-treated microplates (Eppendorf AG, Hamburg, Germany), seeded at 15,000 cells per well, and incubated overnight to allow attachment, followed by treatment with OA and its derivatives for 6, 24 and 48 h at a concentration range of 0.75–100 μM. Control cells were incubated with 0.1% dimethyl sulfoxide (DMSO) solvent (Invitrogen, Thermo Fisher Scientific, Waltham, MA, USA) in a complete growth medium.

### 4.3. Evaluation of Cell Metabolic Activity of Cells Treated with OA and Its Derivatives with the MTT Assay

After the treatment time specified in the experiment conditions, the post-culture medium was discarded, cells were rinsed with sterile phosphate-buffered saline (PBS; Gibco, Thermo Fisher Scientific, Waltham, MA, USA) solution, and a freshly prepared 0.5 mg/mL 3-(4,5-dimethylthiazol-2-yl)-2,5-diphenyl tetrazolium bromide in complete growth medium (MTT reagent; Sigma-Aldrich, St. Luis, MO, USA) was added to the culture. Plates were incubated for 3 h in the CO**_2_** incubator under the above-mentioned conditions. Subsequently, the MTT reagent was gently decanted, and the formed formazan crystals were dissolved in DMSO (Invitrogen, Thermo Fisher Scientific, Waltham, MA, USA). The absorbance was measured with Infinite**^®^** M200 spectrophotometer (Tecan Group Ltd., Mannedorf, Switzerland) at λ = 540 nm. The experiments were conducted in triplicate, utilizing cells from different cell passages.

### 4.4. Evaluation of the Cells Number after Exposure to OA and Its Derivatives with the Sulforhodamine B (SRB) Assay

After the treatment time specified in the experimental conditions, cells were fixed with trichloroacetic acid (TCA; Sigma-Aldrich, St. Luis, MO, USA) at a final concentration of 12.5% and incubated for 1 h at 4°C, followed by gentle rinsing with cold water and drying. Subsequently, a freshly prepared solution of 0.04% SRB (Sigma-Aldrich, St. Luis, MO, USA) in 1% acetic acid (Avantor Performance Materials Poland, Gliwice, Poland) was added and left at room temperature for 30 min. The unbound dye was removed using 1% acetic acid. The protein-bound SRB was solubilized in 10 mM Tris base solution (BioShop, Burlington, Ontario, Canada), pH 10.5. The absorbance, proportional to the protein content, was measured using the Infinite**^®^** M200 spectrophotometer (Tecan Group Ltd., Mannedorf, Switzerland) at λ = 520 nm. The experiments were performed in triplicate, utilizing cells from different cell passages.

### 4.5. Statistical Analysis

Descriptive data were shown as a mean and a standard deviation (± SD). Distribution of the data was tested with the Shapiro–Wilk normality test, and the homogeneity of variances were analyzed by Leven’s test. One-way analysis of variance ANOVA was used for multiple comparison procedure, and the post-hoc Tukey test was used for evaluation of differences between control and study groups. Values with *p* ≤ 0.05 were considered to be statistically significant. Data were analyzed using MS Excel 2016 (Microsoft Co, USA) and Statistica v.13.3 (Tibco Software Inc., Palo Alto, CA, USA).

## Data Availability

Not applicable.
